# In silico analysis of several frequent SLX4 mutations appearing in human cancers

**DOI:** 10.17912/micropub.biology.001216

**Published:** 2024-05-17

**Authors:** Korey Bosart, Ruben C Petreaca, Renee A Bouley

**Affiliations:** 1 James Comprehensive Cancer Center, The Ohio State University, Columbus, Ohio, United States; 2 Molecular Genetics, The Ohio State University at Marion, Marion, Ohio, United States; 3 Chemistry and Biochemistry, The Ohio State University at Marion, Marion, Ohio, United States

## Abstract

SLX4 is an interactor and activator of structure-specific exonuclease that helps resolve tangled recombination intermediates arising at stalled replication forks. It is one of the many factors that assist with homologous recombination, the major mechanism for restarting replication. SLX4 mutations have been reported in many cancers but a pan cancer map of all the mutations has not been undertaken. Here, using data from the Catalogue of Somatic Mutations in Cancers (COSMIC), we show that mutations occur in almost every cancer and many of them truncate the protein which should severely alter the function of the enzyme. We identified a frequent R1779W point mutation that occurs in the SLX4 domain required for heterodimerization with its partner, SLX1. In silico protein structure analysis of this mutation shows that it significantly alters the protein structure and is likely to destabilize the interaction with SLX1. Although this brief communication is limited to only *in silico* analysis, it identifies certain high frequency SLX4 mutations in human cancers that would warrant further *in vivo* studies. Additionally, these mutations may be potentially actionable for drug therapies.

**Figure 1. Pan-cancer analysis of SLX4 mutations:**
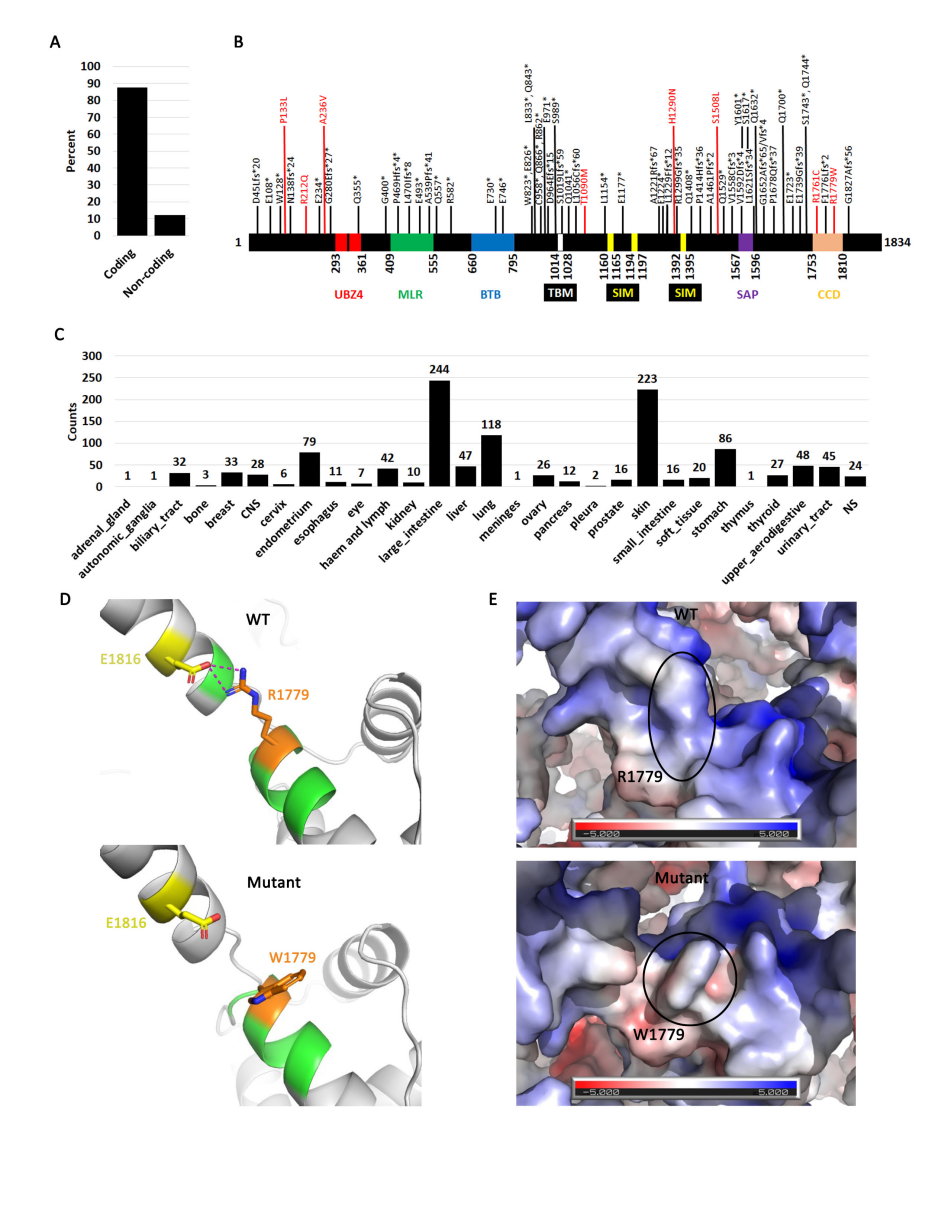
**A** . SLX4 mutations in all cancers are primarily coding. **B** . A map of SLX4 showing the relevant domains. The diagram was adapted from (Payliss et al., 2021). Shown on the diagram are truncating mutations (black) and high frequency mutations (>5) in red. **C** . Distribution of coding mutations (silent, missense, nonsense, InDel) by cancer type. **D.** (Top) Model showing location of WT R1779 residue (orange) interacting with the side chain of E1816 (yellow) through a salt-bridge (shown in dashed magenta lines). (Bottom) A model of the W1779 (orange) mutation with the loss of the E1816 (yellow) salt-bridge interaction. **E** . (Top) Electrostatic surface potential of the WT protein where the location of the R1779 is circled. (Bottom) Electrostatic surface potential of the R1779W mutant where the location of the W1779 residue is circled. Blue represents a basic or positive charge, red is an acidic or negative charge, white is neutral.

## Description

In human cells, *H* omologous *R* ecombination (HR) is the primary mechanism for repair and restart of replication forks (rev in (Chakraborty, Schirmeisen, & Lambert, 2023) ). It also contributes to repair of DNA *D* ouble *S* trand *B* reaks (DSBs) primarily in S-phase and mitosis (rev in (J. Li et al., 2019; Tan et al., 2023)). Stalled replication forks produce all forms of complex branched DNA structures and require a plethora of nucleases and helicases to resolve them (rev in (Pasero & Vindigni, 2017) ). SLX4 was identified in a yeast synthetic lethal screen with Sgs1 (human ReqQ helicase) (Mullen, Kaliraman, Ibrahim, & Brill, 2001) **.** RecQ had been previously identified in human cells (Seki et al., 1994) but it was work in yeast that first established it to be involved in DNA damage repair in S-phase (Murray, Lindsay, Munday, & Carr, 1997) . The synthetic lethality between SLX4 and Sgs1/RecQ suggest that they form two distinct pathways for resolving recombination intermediates.

SLX4 functions to clean up branched recombination intermediates (rev in (Guervilly & Gaillard, 2018) ) to rescue stalled replication forks (Fricke & Brill, 2003) . It functions as a heterodimer complex with Slx1 (Coulon et al., 2004) . The major function of SLX4 is to act as a scaffold to recruit numerous factors to stalled replication forks (rev in (Payliss, Patel, Sheppard, & Wyatt, 2021) ) and several domains within the SLX4 protein sequence are required for this recruitment function (Fig.1C). The mismatch repair heterodimer MSH2-MSH3 interacts with the N-terminus of SLX4 (F. Li et al., 2013; F. Li et al., 2008; Young et al., 2020). Two ubiquitin binding domains (UBZ4) are required for recruitment of SLX4 to DNA damage sites (Guervilly et al., 2015; Katsuki et al., 2021; Lachaud et al., 2014) . The MLR domain also helps with SLX4 DNA damage site recognition but has an additional crucial function in interacting and recruiting XPF-ERCC1 (Hoogenboom, Boonen, & Knipscheer, 2019) , a complex with primary roles in nucleotide excision repair but also acting on DSBs and stalled forks (rev in (Faridounnia, Folkers, & Boelens, 2018) ). The BTB domain is required for dimerization primarily through the F681 and F704 residues and disruption of this dimerization affects telomere maintenance and sensitivity to crosslinking agents (Yin et al., 2016) . The TBM domain also facilitates its telomere associated function by recruiting the telomere repeat binding factor (TRF) (Wan et al., 2013) . The SUMO interacting motifs (SIMs) are required for forming SLX4 aggregates on chromatin which facilitates removal of DNA interacting proteins (Alghoul et al., 2023) . The SAP domain positions SLX4 at the branched intermediate so that it can access the 5’ flap and process it (Xu et al., 2021) . Certain residues within the vicinity of this domain (T1544, T1561, T1571) are phosphorylated by CDK1/Cyclin B which induces a conformation change in the SAP domain required for recruitment of MUS81-EME1 (Payliss et al., 2022) . MUS81-EME1 constitute a structure specific endonuclease dimer (rev in (Rass, 2013) ). Finally, the CCD domain interacts with and activates SLX1 (Xu et al., 2021) .

SLX4 mutations have been identified in Fanconi anemia and the gene is also known as FANCP ( (Garner & Smogorzewska, 2011; Kim et al., 2011; Su & Huang, 2011) , rev in (Kim, 2014) ). Both germline and spontaneous (somatic) mutations have been identified. Germline mutations predispose patients to breast and ovarian cancers (Adamson et al., 2023; Alter & Best, 2020; Catucci et al., 2012; de Garibay et al., 2013; Fernandez-Rodriguez et al., 2012; Ke et al., 2023) as well as some neuro-endocrine tumors (Riechelmann et al., 2023) . Spontaneous mutations appear in a variety of cancers including colorectal (Zhunussova et al., 2019) , cervical (Lu, Fan, Huo, Feng, & Wang, 2024) , liver (Perez et al., 2023) , breast (Felix et al., 2022) as well as others. Certain small molecule inhibitors that disrupt SLX4 interactions with its associated proteins have been shown to be good therapeutic agents (rev in (Jordheim, 2023) ).

Here we present a pan-cancer analysis of SLX4 mutations using data from the Catalogue of Somatic Mutations in Cancers (COSMIC). We also use *in silico* three-dimensional protein structure analysis to understand how high frequency SLX4 mutations affect its function.

COSMIC reports both coding (protein sequence) and non-coding (5’ and 3’ UTRs and intronic) mutations. Coding mutations are likely to have a greater effect on destabilization of enzyme function than non-coding ones. An SLX4 pan-cancer classification of these mutations shows that the highest percentage of them are coding and will change the protein sequence. Interrogation of NCBI (www.ncbi.nlm.nih.gov) and the human genome browser (https://genome.ucsc.edu/) reveals that the SLX4 primary transcript (including UTRs and introns) is 30,426 nucleotides while the processed mature mRNA is 7,315 nucleotides. Thus, the coding sequence represents 24% of the gene. The fact that there is a higher incidence in the coding regions may suggest that coding mutations are selected for in cancer tissues ( **Fig.1A** ). Therefore, coding SLX4 mutations are endemic in cancer cells and loss of function of this enzyme is likely to contribute to cellular transformation and immortalization.

We next wanted to see where these coding mutations mapped within the sequence of SLX4. Although coding mutations fall into missense, nonsense, InDels, and silent we chose only those that have the most drastic effect on protein/enzyme function. Nonsense as well as 1 or 2 base pair insertions and deletions which produce frameshifts are the most deleterious mutations ( **Fig.1B** **black labels** ). We found that truncating mutations distribute throughout the sequence of the gene but there appears to be a higher concentration around the TBM domain and within the C-terminal region of the protein. COSMIC lists SLX4 mutations from 1381 samples of which only 160 (11.6%) have information on zygosity. None of the truncating mutations are heterozygous (for samples where zygosity is listed). To further explore SLX4 biallelic inactivation status, we used the COSMIC *Co* py *N* umber *An* alysis (CONAN) tool (Forer et al., 2010) . Rather than point mutations, CONAN lists whole gene alterations such as amplification, homozygous deletion (deletion both alleles), and loss of heterozygosity (deletion of one allele). Such large-scale allele alterations were identified in 49 samples from The Cancer Genome Atlas (TCGA) studies. Of these, 24 (49%) were amplifications, 8 (16.3%) were homozygous deletions and 17 (34.7%) were loss of heterozygosity. Thus, this suggests that although SLX4 biallelic inactivation does occur, it is rare.

A partitioning of coding mutations by cancer type shows that they appear in all cancers ( **Fig.1C** ). The observation that some cancers have more mutations than others (e.g. skin and large intestine) is explained by the fact that more data is available for these cancers. Thus, **Fig.1C** should be interpreted with this caveat and not that SLX4 mutations are likely to occur in some cancers more than others.

In cancer genetics, there is an aim to classify mutations as driver or passenger. Driver mutations are considered to greatly influence cancer initiation and progression while passenger mutations may make peripheral contributions or none at all (e.g. background mutations). A recent report identified all human cancer driver genes (Martinez-Jimenez et al., 2020) but SLX4 was not listed as a driver gene. This is generally interpreted to mean that mutations in SLX4 are not likely to have a great impact on cellular transformation and immortalization. This is not unexpected considering that several other nucleases and helicases exist that may assist with SLX4 function (Nickoloff et al., 2023) . However, given the function of SLX4 and the fact that SLX4 mutations occur in nearly all cancers it is not unreasonable to conclude that mutations in this gene also contribute to cancer development.

We next turned our attention to high frequency point mutations which occur in five samples or more. Juul et al (Juul, Nielsen, Juul, Feuerbach, & Pedersen, 2021) define highly mutated hotspots as 4 or more mutations but because COSMIC reports both data from primary cancers as well as cell line which are more transformed, we increase the stringency to 5 or more mutations at same position. We identified eight high frequency mutations (P133L, R212Q, A236V, T1090M, H1290N, S1508L, R1761C, and R1779W) ( **Fig.1B** , red labels). To understand how these mutations affect the protein structure, we used *in silico* 3D modeling of the SLX4 structure. A wild-type structural model for the SLX4 protein was obtained from AlphaFold (Varadi et al., 2022; Zhu, Shenoy, Kundrotas, & Elofsson, 2023) because there are no available experimentally derived full-length structures for the human protein from the Protein Data Bank. This model contained large sections of the protein that were highly disordered, which is consistent with previous studies (Payliss et al., 2021) **.** Computational analysis of the 8 mutations was structured into a three-prong approach that consisted of side-chain interaction analyses, protein stability predictions, and electrostatic surface potential calculations (Stan et al., 2024) .

The side-chain interaction analyses were based on the polar contacts with the side chains of the WT and mutant residues, which are important for the tertiary structure. This analysis was performed for all 8 high frequency mutations using PyMOL. Of these 8 residues, 5 were located in disordered regions (P133, A236, T1090, H1290, and S1508) of the AlphaFold model and thus these mutations would not be expected to significantly effect protein structure. Mutations that reduced the number of polar contacts in comparison to WT were identified as being significant. Only one mutation (R1779W) out of the 8 was identified as a mutation that reduced polar interactions (hydrogen bonds, dipole-dipole, and salt bridges) relative to WT. Calculation of electrostatic surface potential was the second analysis performed on the 8 mutations. The electrostatic mapping of the mutations allows for electrostatic alterations to be identified according to a visual analysis of any color changes as well as of any size or shape changes in the protein surface area. 3 out of the 8 mutations (R212Q, R1761C, R1779W) displayed noticeable differences in protein surface area and/or visual electrostatic color differences. Mutations were also examined using a CUPSAT analysis that determined their effect on protein stability represented as a ΔΔG value (Parthiban, Gromiha, & Schomburg, 2006) . The ΔΔG value represents the difference in the unfolding ΔG value for the WT protein and mutant protein. Negative values indicate destabilization of protein structure while positive values indicate stabilization of structure. For this analysis, 3 mutations that had a ΔΔG value of less than -1 kcal/mol (T1090M, R1761C, and R1779W) and 1 mutation had a ΔΔG value greater than 1 kcal/mol (S1508L).

The compilation of the three-prong analyses data enabled the classification and identification of the most significant high frequency mutations affecting protein structure. The data was compiled in an overlapping format where mutations were classified on their significance based on the number of analyses that they were identified as significant across the three total analyses. The total list of 8 mutations was able to be narrowed down to a single mutation that could be identified as the most significant high frequency mutation to affect protein structure in all 3 analyses, R1779W ( **Figure 1D, E, F** ). The R1779W mutation is found in the conserved C-terminal domain (CCD) of SLX4, which has been shown to be critical for interacting with SLX1 (Payliss et al., 2021) . Of all the point mutations for which zygosity is reported, only the P133L high frequency mutation is homozygous. This mutation is from a head and neck squamous cell carcinoma (Seiwert et al., 2015) . These data show that SLX4 haploinsufficiency may be sufficient to contribute to cellular transformation.

Our analysis shows that although SLX4 is not a driver gene, it is highly mutated in most cancers. This suggests that it contributes to cellular transformation and cancer. This study was limited to *in silico* analysis of SLX4 mutations and further *in vivo* experimental evidence is warranted to understand how mutations such as the R1779W affect enzyme function. Nevertheless, we identified a potentially actionable residue in SLX4.

## Methods

*Data acquisition.* All data were downloaded from the Catalogue of Somatic Mutations in Cancers version 99 as an .csv file (Tate et al., 2019) . The COSMIC Copy Number Analysis (CONAN) tool (Forer et al., 2010) was used to extract changes in gene copy number. Figures were made in Photoshop.

*Protein Structure Computational Analysis of Mutations.* A model of the human SLX4 protein structure was obtained from AlphaFold (Jumper et al., 2021) . The mutagenesis function of the program PyMOL was utilized to produce desired amino acid mutations. PyMOL was also used for the observation and analysis of side chain polar interactions within a 4 Å radius for the wild-type and mutant residues. Electrostatic surface potential maps of the selected driver mutations were produced using the APBS electrostatics plugin of the PyMOL program. CUPSAT analysis using the BRENDA enzyme database was performed to determine if the mutations effected protein stability. The full-length SLX4 AlphaFold model was too large for CUPSAT analysis, so the model was divided into three smaller regions containing the 8 mutated driver residues that were analyzed separately: N-terminal region (133-236), central region (1090-1290), and C-terminal region (1508-1779). Mutation(s) that showed an effect in all 3 of these separate analyses ( **Table 1** ) were then identified for further analysis.

## Reagents

**Table:** 

**Table 1. Three-pronged approach for analyzing effect of mutations on SLX4 structure.**				
**Mutation**	**Region/Domain of SLX4 protein**	**Analysis 1: Change in Polar Interaction**	**Analysis 2: Electrostatic Change**	**Analysis 3: Predicted** ΔΔ **G**
P133L	Disordered N-terminus	No	Neutral to Slightly Acidic	-0.53
R212Q	Alpha helix in N-terminus	No	Slightly Basic to Slightly Acidic	-0.3
A236V	Disordered N-terminus	No	Neutral to Neutral	0.68
T1090M	Disordered linker between TBM and SIM1 domains	No	Slightly Basic to Slightly Basic	-2.11
H1290N	Disordered linker between SIM3 and SAP domains	No	Slightly Acidic to Slightly Acidic	0.11
S1508L	Disordered linker between SIM3 and SAP domains	No	Slightly Acidic to Slightly Acidic	1.14
R1761C	CCD domain	No	Slightly Basic to Neutral	-1.79
R1779W	CCD domain	Decrease	Slightly Basic to Neutral	-2.51
